# Rare and common coding variants in lipid metabolism-related genes and their association with coronary artery disease

**DOI:** 10.1186/s12872-024-03759-5

**Published:** 2024-02-09

**Authors:** Wei Li, Yongyi Wang, Ritai Huang, Feng Lian, Genxing Xu, Weijun Wang, Song Xue

**Affiliations:** Department of Cardiovascular Surgery, Renji Hospital, Shanghai Jiaotong University School of Medicine, Shanghai, 200127 China

**Keywords:** CAD (coronary artery disease), Targeted sequencing, Rare variants, Common variant

## Abstract

**Background:**

Coronary artery disease (CAD) is a complex disease that is influenced by environmental and genetic factors. In this study, we aimed to investigate the relationship between coding variants in lipid metabolism-related genes and CAD in a Chinese Han population.

**Methods:**

A total of 252 individuals were recruited for this study, including 120 CAD patients and 132 healthy control individuals. Rare and common coding variants in 12 lipid metabolism-related genes (*ANGPTL3*, *ANGPTL4*, *APOA1*, *APOA5*, *APOC1*, *APOC3*, *CETP*, *LDLR*, *LIPC*, *LPL*, *PCSK9* and *SCARB1*) were detected via next-generation sequencing (NGS)-based targeted sequencing. Associations between common variants and CAD were evaluated by Fisher’s exact test. A gene-based association test of rare variants was performed by the sequence kernel association test-optimal (SKAT-O test).

**Results:**

We found 51 rare variants and 17 common variants in this study. One common missense variant, LIPC rs6083, was significantly associated with CAD after Bonferroni correction (OR = 0.47, 95% CI = 0.29–0.76, *p* = 1.9 × 10^− 3^). Thirty-three nonsynonymous rare variants were identified, including two novel variants located in the *ANGPTL4* (p.Gly47Glu) and *SCARB1* (p.Leu233Phe) genes. We did not find a significant association between rare variants and CAD via gene-based analysis via the SKAT-O test.

**Conclusions:**

Targeted sequencing is a powerful tool for identifying rare and common variants in CAD. The common missense variant LIPC rs6083 confers protection against CAD. The clinical relevance of rare variants in CAD aetiology needs to be investigated in larger sample sizes in the future.

**Supplementary Information:**

The online version contains supplementary material available at 10.1186/s12872-024-03759-5.

## Introduction

Coronary artery disease (CAD) is a common chronic inflammatory disease that remains the leading cause of death worldwide [1]. It was estimated that 700,000 people die from CAD in China every year [2]. Lipid disorders are common conditions that involve abnormal levels of lipids in the blood. These disorders are a significant risk factor for the development of CAD. High levels of low-density lipoprotein (LDL) cholesterol, known as “bad” cholesterol, and low levels of high-density lipoprotein (HDL) cholesterol, known as “good” cholesterol, are associated with an increased risk of CAD. Elevated triglyceride levels can promote plaque formation, and high levels of lipoprotein(a) have been shown to increase the risk of CAD. In addition to conventional risk factors such as hypertension, dyslipidaemia, diabetes, obesity and smoking, genetic factors also play an important role in CAD pathogenesis. To reduce the occurrence of CAD, identifying biomarkers responsible for CAD aetiology is important [3].

Genome-wide association studies have identified many variants associated with CAD [4–7]. GWASs focus on common variants, and these susceptibility variants are always located within intronic or intergenic regions with relatively small effects. Common and rare variants in the coding region that might also be associated with CAD are generally missed.

Due to the increase in throughput and decrease in costs, next-generation sequencing (NGS)-based technology has been shown to be a powerful tool for identifying novel causal mutations associated with Mendelian diseases [8]. Targeted sequencing is a rapid and cost-effective way to detect known and novel variants in selected sets of genes or genomic regions and has been shown to be an efficient technique for screening variants in complex diseases [9, 10]. Targeted sequencing of a subset of genes generates results with quality identical to that of Sanger sequencing [11]. Four rare variants in the coding region of apolipoprotein C3 (*APOC3*) that disrupt *APOC3* function were found to be associated with lower plasma triglyceride levels and a reduced risk of coronary heart disease [12]. Dewey et al. showed that patients carrying inactivating variants in *ANGPTL4* had lower triglyceride levels and a lower risk of CAD than noncarriers [13]. Compound heterozygosity for two distinct nonsense variants in *ANGPTL3* results in decreased plasma LDL cholesterol levels and familial combined hypolipidaemia [14]. Rare alleles at *LDLR* and *APOA5* confer risk for early-onset myocardial infarction [15]. Rare nonsynonymous variants can facilitate the exploration of disease pathogenesis and provide supportive evidence for putative drug targets for novel therapies.

In this study, we conducted targeted sequencing of 12 genes involved in lipoprotein metabolism to investigate both common and rare variants and their association with CAD. We aimed to identify genetic risk factors that confer susceptibility to CAD in the Chinese Han population, thereby shedding light on the pathogenesis of CAD.

## Materials and methods

### Study population

A total of 120 CAD patients and 132 non-CAD control individuals were recruited from Renji Hospital between 2016 and 2020. All of the participants were adults who signed an informed consent form. The average age of the participants was 64.60 ± 9.58 years. All the participants were unrelated Chinese Han individuals. This study was approved by the Medical Ethics Committee of Renji Hospital and complied with the principles set forth by the Declaration of Helsinki. The diagnostic criteria for CAD patients were defined as follows: at least one of the major segments of coronary arteries (right coronary artery, left circumflex, or left anterior descending arteries) with ≥ 50% organic stenosis based on coronary angiography. The clinical characteristics of the CAD patients are summarized in Supplementary Table 1. Non-CAD control individuals were defined as those free from coronary lesions (angiography normal). Individuals with incomplete information were excluded. A 5 ml peripheral blood sample was collected from each subject.

### Targeted sequencing

Genomic DNA was extracted using a TianGen DNA Extraction Kit (TianGen Ltd., Beijing, China) following the standard protocol. The DNA concentration and quality were measured using a NanoDrop spectrophotometer (Thermo Scientific, USA). All the purified DNA was stored at -80 °C, and 50ng DNA was used for PCR amplification. We selected lipid metabolism-related genes significantly associated with CAD based on existing genome-wide association studies and case‒control studies. We generated a multiplex PCR panel to capture the coding region of lipid metabolism-related genes. After pre-experimental adjustments, 12 genes were ultimately retained for subsequent experiments (*ANGPTL3*, *ANGPTL4*, *APOA1*, *APOA5*, *APOC1*, *APOC3*, *CETP*, *LDLR*, *LIPC*, *LPL*, *PCSK9* and *SCARB1*). All the genes were shown to be associated with CAD in previous studies (Table 1). PCR primers (Supplementary Table 2) were designed using Oligo 6.0 and synthesized by Shanghai Free Biotechnology Co., Ltd. (Shanghai, China). Coding regions of the target gene were captured by multiplex PCR followed by adaptor addition. The final panel consisted of 203 amplicons with an average size of 250 bp. Paired-end sequencing (2 × 150) was performed with Illumina NovaSeq sequencing instruments (Novogene, Beijing, China).

**Table 1:** The 12 genes selected for the NGS custom gene panel

Gene	Location	Ref seq no.	No. of coding exons	Transcript length (bp)	Protein length (aa)	Ref
*ANGPTL3*	1p31.3	NM_014495.4	7	2926	460	[16]
*ANGPTL4*	19p13.2	NM_139314.3	7	1872	406	[13, 17]
*APOA1*	11q23.3	NM_000039.3	4	899	267	[18]
*APOA5*	11q23.3	NM_001371904.1	3	1881	366	[19, 20]
*APOC1*	19q13.32	NM_001645.5	4	514	83	[18]
*APOC3*	11q23.3	NM_000040.3	4	535	99	[12, 21, 22]
*CETP*	16q13	NM_000078.3	16	1691	493	[23–25]
*LDLR*	19p13.2	NM_000527.5	18	5173	860	[26]
*LIPC*	15q21.3	NM_000236.3	9	2559	499	[27]
*LPL*	8p21.3	NM_000237.3	10	3565	475	[26, 28]
*PCSK9*	1p32.3	NM_174936.4	12	3637	692	[26, 28]
*SCARB1*	12q24.31	NM_005505.5	13	3405	509	[29, 30]

### Variant analysis

Nonsynonymous exonic variants were identified by BWA (version 0.7.17) and SAMtools (version 1.9) according to the following quality control criteria: (1) at least 50× coverage; (2) Q-score > 30; and (3) at least 40% variant frequency. Variant annotation was performed on GRCh38.p13. Variations that were absent or had a minor allele frequency < 0.01 in the public database (dbSNP build 155; Exome Aggregation Consortium v1.0; 1000 Genomes Project phase 3; and Genome Aggregation Database v3.1.1) were regarded as rare variants. Common variants were defined as having a minor allele frequency > 0.05, and low-frequency variants were defined as having a minor allele frequency between 0.01 and 0.05. Associations between common variants and CAD were determined using a standard Fisher’s exact test with default parameters. The *p* value was adjusted to 2.9 × 10^− 3^ by Bonferroni correction (0.05/17). A gene-based association test of rare variants was performed using the sequence kernel association test-optimal (SKAT-O) test [31]. Pathogenicity prediction of the missense variant was performed with SIFT v4.0.3 (http://provean.jcvi.org/index.php) and PolyPhen-2 version 2.2.3 (http://genetics.bwh.harvard.edu/pph2/index.shtml) [32, 33]. Variants were classified into five classes (benign, likely benign, uncertain significance, likely pathogenic, and pathogenic) based on the ACMG guidelines [34]. Multiple sequence alignment was performed using the Clustal method [35].

## Results

We screened all the exons of 12 lipid metabolism-related genes (*ANGPTL3, ANGPTL4, APOA1, APOA5, APOC1, APOC3, CETP, LDLR, LIPC, LPL, PCSK9 and SCARB1*) and their flanking sequences in 120 CAD patients and 132 control individuals. A total of 75 variants were identified after quality control, including 51 rare variants (MAF < 0.01), 7 low-frequency variants (MAF: 0.01–0.05) and 17 common variants (MAF > 0.05).

Seventeen common variants were identified, including 9 synonymous variants and 8 nonsynonymous variants. Common variant association analysis revealed that 4 variants located in the CETP and LIPC genes were nominally associated with CAD (*p* value < 0.05) (Table 2). Nevertheless, after Bonferroni correction (*p* value < 2.9 × 10^− 3^), the missense variant LIPC rs6083 remained significantly associated (*p* value = 1.9 × 10^− 3^).

**Table 2:** Association analysis of common variants

Gene	chr	Position	Ref/Alt	rsID	Effect	MAF	OR (95% CI)	*p*
ANGPTL4	19	8,371,280	C/T	rs1044250	missense variant	0.057/0.068	1.210 (0.579 ~ 2.528)	0.611
APOA5	11	116,790,772	G/A	rs3135507	missense variant	0.110/0.097	0.864 (0.419 ~ 1.783)	0.693
CETP	16	56,982,180	G/A	rs5882	missense variant	0.439/0.534	1.463 (1.010 ~ 2.118)	0.044
LDLR	19	11,113,589	A/G	rs5930	synonymous variant	0.327/0.392	1.329 (0.842 ~ 2.097)	0.221
LDLR	19	11,116,124	C/T	rs5929	synonymous variant	0.346/0.292	0.779 (0.535 ~ 1.134)	0.192
LDLR	19	11,116,926	C/T	rs688	synonymous variant	0.146/0.144	0.985 (0.599 ~ 1.618)	0.952
LDLR	19	11,120,205	T/C	rs5925	synonymous variant	0.218/0.187	0.825 (0.518 ~ 1.313)	0.417
LIPC	15	58,541,794	A/G	rs6078	missense variant	0.278/0.280	1.013 (0.684 ~ 1.499)	0.950
LIPC	15	58,542,542	G/T	rs690	synonymous variant	0.292/0.275	0.919 (0.611 ~ 1.380)	0.682
LIPC	15	58,545,758	A/G	rs6082	synonymous variant	0.479/0.402	0.729 (0.512 ~ 1.038)	0.079
LIPC	15	58,545,811	G/A	rs6083	missense variant	0.221/0.117	0.469 (0.290 ~ 0.761)	1.9E-03
LIPC	15	58,545,839	C/G	rs6084	synonymous variant	0.117/0.057	0.456 (0.237 ~ 0.877)	0.016
LIPC	15	58,568,764	A/C	rs6074	synonymous variant	0.199/0.312	1.822 (1.167 ~ 2.845)	7.9E-03
LPL	8	19,962,213	C/G	rs328	stop gained	0.092/0.083	0.895 (0.471 ~ 1.702)	0.734
PCSK9	1	55,039,880	-/CTG	rs35574083	inframe insertion	0.067/0.099	1.529 (0.799 ~ 2.927)	0.197
PCSK9	1	55,039,995	C/T	rs11583680	missense variant	0.083/0.102	1.253 (0.683 ~ 2.299)	0.465
SCARB1	12	124,800,202	C/T	rs5888	synonymous variant	0.213/0.261	1.311 (0.867 ~ 1.983)	0.198

Chr, chromosome; Position, genomic position based on GRCh38.p13; Ref/Alt, reference allele/alternative allele; MAF, minor allele frequency of case/control; OR, odds ratio; CI, confidence interval; p, Fisher’s *p* value

To reveal the potential burden of rare variants in the CAD group compared to the control group, we conducted a gene-based association analysis using the SKAT-O test. However, no significant difference was found between the CAD patients and healthy control individuals (Table 3).

**Table 3:** Gene-based sequence kernel association test-optimal (SKAT-O) test of rare variants

Gene	Number of Variants	*p* Value
ANGPTL3	2	0.913
ANGPTL4	5	0.202
APOA1	5	0.926
APOA5	3	0.117
APOC1	2	0.654
CETP	4	0.821
LDLR	6	0.897
LIPC	6	0.722
LPL	4	0.059
PCSK9	8	0.594
SCARB1	6	0.681

A total of 33 coding nonsynonymous variants, including 32 missense variants and one 7 bp duplication variant, were discovered in 12 gene regions (Table 2). All of these variants were heterozygous variants (Table 4). Of all the rare nonsynonymous variants, 18 were identified only in the CAD group, 12 were identified only in the control group, and 3 were identified in both the CAD and control groups. Of all the rare synonymous variants, 3 were identified only in the CAD group, 10 were identified only in the control group, and 5 were identified in both the CAD and control groups.

Two novel missense variants discovered in this study were not found in the ExAC, gnomAD or dbSNP databases, and both were found in the CAD cohort. One single nucleotide variant in the *ANGPTL4* gene that introduces a missense variant at position 47, resulting in the amino variant p.Gly47Glu (GGA-GAA, located in the first exon of *ANGPTL4* at nucleotide 8,364,461 on chromosome 19). The other single-nucleotide variant in the *SCARB1* gene that introduces a missense variant at position 233, resulting in the amino variant p.Leu233Phe (CTC-TTC, located in the fifth exon of *SCARB1* at nucleotide 124,811,899 on chromosome 12). We also identified novel alleles at 4 existing SNVs in the dbSNP database. Two novel variants and four novel alleles were validated by bidirectional Sanger sequencing and demonstrated 100% concordance (Supplementary Fig. 1).

Variant pathogenicity analysis was performed using SIFT and Polyphen-2. Twelve variants were predicted to be deleterious by SIFT, and 18 were predicted to be possibly damaging or probably damaging by PolyPhen-2. Ten variants were predicted to be tolerated by SIFT and benign by PolyPhen-2, indicating that these variants are not pathogenic. Eight variants were predicted to be deleterious/damaging in both programs. Twenty-two variants were predicted to be damaging or deleterious in at least one program. According to the standards and guidelines of the ACMG, p.Asp168Asn in the LDLR gene was classified as a likely pathogenic variant, while the rest were classified as having uncertain significance or benign.

**Table 4:** Rare nonsynonymous variants identified in this study

Gene	chr	pos	Ref/Alt	DNA	AA	novel	Effect	dbSNP ID	MAF (gnomAD)	Number of variants		SIFT	sift_class	PolyPhen	polyphen_class	ACMG
										case	control					
ANGPTL3	1	62,598,787	T/C	c.587T > C	p.Ile196Thr		missense variant	rs201826477	0.00001974	0	1	0.01	deleterious	0.998	probably damaging	VUS
ANGPTL3	1	62,598,796	T/C	c.596T > C	p.Ile199Thr		missense variant	rs112068132	0.00004609	1	1	0	deleterious	0.197	benign	VUS
ANGPTL4	19	8,364,461	G/A	c.140G > A	p.Gly47Glu	yes	missense variant		/	1	0	0.003	deleterious	1	probably damaging	VUS
ANGPTL4	19	8,371,453	G/A	c.970G > A	p.Val324Ile		missense variant	rs200918932	0.00006572	1	0	0.1	tolerated	0.023	benign	VUS
ANGPTL4	19	8,373,780	A/G	c.1115 A > G	p.Gln372Arg		missense variant	rs756440132	0.0001248	0	1	0.3	tolerated	0.003	benign	VUS
APOA1	11	116,837,116	G/C	c.85 C > G	p.Gln29Glu		missense variant	rs1254205437(G/T)	/	1	0	0.11	tolerated	0.005	benign	VUS
APOA1	11	116,837,080	C/A	c.121G > T	p.Val41Leu		missense variant	rs201148448	0.000006569	1	1	0.07	tolerated	0.001	benign	VUS
APOA1	11	116,837,053	C/T	c.148G > A	p.Gly50Ser		missense variant	rs28931574	0.00000657	0	1	0.08	tolerated	0.998	probably damaging	VUS
APOA1	11	116,836,173	G/T	c.439 C > A	p.Arg147Ser		missense variant	rs1591330063(G/C)	/	1	0	0.649	tolerated	0.101	benign	VUS
APOA5	11	116,791,670	C/A	c.77G > T	p.Gly26Val		missense variant	rs548745995	/	1	0	0.19	tolerated	0.642	possibly damaging	VUS
APOA5	11	116,790,166	G/C	c.1063 C > G	p.Leu355Val		missense variant	rs556600766(G/A)	/	1	0	0.005	deleterious	0.124	benign	VUS
APOC1	19	44,916,230	T/TCTTGGAT	c.99_105dup	p.Lys36LeufsTer3		frameshift variant	rs767630355	0.00001978	0	1	-	-	-	-	VUS
APOC1	19	44,916,292	G/A	c.161G > A	p.Arg54His		missense variant	rs369438021	0.00003289	1	0	0.6	tolerated	0	benign	VUS
CETP	16	56,962,013	C/G	c.34 C > G	p.Leu12Val		missense variant	rs1460617147(C/T)	/	0	1	0.294	tolerated	0.772	possibly damaging	VUS
CETP	16	56,969,935	G/A	c.461G > A	p.Arg154Gln		missense variant	rs184615182	0.00001971	1	0	0.37	tolerated	0.003	benign	VUS
LDLR	19	11,105,250	G/A	c.344G > A	p.Arg115His		missense variant	rs201102461	0.00008546	0	1	0.04	deleterious	0.102	benign	VUS
LDLR	19	11,105,408	G/A	c.502G > A	p.Asp168Asn		missense variant	rs200727689^a^	0.000006569	1	0	0.03	deleterious	1	probably damaging	LP
LDLR	19	11,105,492	C/G	c.586 C > G	p.Pro196Ala		missense variant	rs1013147010	/	0	1	0.194	tolerated	0.988	probably damaging	VUS
LDLR	19	11,105,505	T/G	c.599T > G	p.Phe200Cys		missense variant	rs879254586	/	1	0	0.18	tolerated	0.779	possibly damaging	VUS
LDLR	19	11,116,900	C/T	c.1747 C > T	p.His583Tyr		missense variant	rs730882109^b^	0.00003286	1	0	0	deleterious	1	probably damaging	VUS
LIPC	15	58,563,522	G/A	c.1187G > A	p.Ser396Asn		missense variant	rs1015457944	0.00000657	0	1	0.26	tolerated	0.725	possibly damaging	VUS
LIPC	15	58,563,665	C/T	c.1330 C > T	p.Arg444Cys		missense variant	rs573340043	0.00004599	1	0	0.06	tolerated	0.809	possibly damaging	VUS
LPL	8	19,951,811	G/A	c.292G > A	p.Ala98Thr		missense variant	rs145657341	0.00006574	1	0	0.04	deleterious	1	probably damaging	VUS
LPL	8	19,954,327	G/A	c.749G > A	p.Arg250His		missense variant	rs750750025	0.00001314	0	1	0.15	tolerated	0.767	possibly damaging	VUS
LPL	8	19,955,927	G/A	c.862G > A	p.Ala288Thr		missense variant	rs1800011	0.00001314	1	0	0	deleterious	0.933	probably damaging	VUS
PCSK9	1	55,046,626	C/T	c.503 C > T	p.Ala168Val		missense variant	rs770592607	0.00001973	0	1	0.33	tolerated	0.003	benign	VUS
PCSK9	1	55,052,650	G/A	c.658G > A	p.Ala220Thr		missense variant splice region variant	rs768795323	0.00001314	1	0	0.03	deleterious	0.313	benign	VUS
PCSK9	1	55,052,698	G/A	c.706G > A	p.Gly236Ser		missense variant	rs149489325	0.00003284	0	1	0.04	deleterious	0.957	probably damaging	VUS
PCSK9	1	55,052,739	C/A	c.747 C > A	p.Ser249Arg		missense variant	rs768846693	0.00001314	1	0	0.01	deleterious	0.996	probably damaging	VUS
PCSK9	1	55,061,420	C/T	c.1727 C > T	p.Pro576Leu		missense variant	rs72646525	0.00005255	1	0	0.19	tolerated	0.011	benign	VUS
SCARB1	12	124,863,717	C/T	c.4G > A	p.Gly2Ser		missense variant	rs4238001	0.08561	0	1	0.1	tolerated	0.994	probably damaging	B
SCARB1	12	124,811,911	T/C	c.685 A > G	p.Ser229Gly		missense variant	rs10396213	0.0001184	1	1	0.09	tolerated	0.41	benign	VUS
SCARB1	12	124,811,899	C/T	c.697G > A	p.Leu233Phe	yes	missense variant		/	1	0	0.147	tolerated	0.903	possibly damaging	VUS

chr, chromosome; pos, genomic position based on GRCh38.p13; Ref/Alt, reference allele/alternative allele; MAF, minor allele frequency in the Genome Aggregation Database; ACMG, variant classification: pathogenic (P), likely pathogenic (LP), variant of uncertain significance (VUS), likely benign (LB) and benign (B)

^a^Annotated as Pathogenic/Likely pathogenic in the ClinVar database (ncbi.nlm.nih.gov/clinvar/variation/183,136/)

^b^Annotated as Conflicting interpretations of pathogenicity in the ClinVar database (ncbi.nlm.nih.gov/clinvar/variation/200,921/)

## Discussion

In the present study, we systemically screened the coding regions of 12 lipid metabolism-related genes in a Chinese cohort of 120 CAD patients and 132 healthy control individuals. NGS based targeted sequencing can identify not only disease-causing variants but also variants of uncertain significance, which can be challenging for genetic counselling.

We found that the missense variant *LIPC* rs6083 was associated with protection from CAD. *LIPC* encodes hepatic triglyceride lipase and participates in the hydrolysis of triglycerides (TGs) and phospholipids [36, 37]. It has been reported that variants in the promoter region of *LIPC* affect HDL-cholesterol levels [38, 39]. Epigenetic analysis revealed that CAD patients had higher *LIPC* DNA methylation levels than healthy control individuals [40]. However, further functional verification is needed to determine whether the missense variant *LIPC* rs6083 affects the pathogenicity of CAD development. Rare variants have a population incidence of < 1% and may not be statistically associated with diseases of interest even in large samples. It was predicted that 27–29% of nonsynonymous variants are neutral or nearly neutral, 30–42% are moderately deleterious, and the remainder are highly deleterious or lethal [41]. Previous studies have shown that rare variants in lipid metabolism genes are associated with CAD. Stitziel et al. reported 37 and 161 loss-of-function variants in 21,980 CAD patients and 158,200 control individuals, respectively. These authors suggested that *ANGPTL3* deficiency is associated with protection from CAD (OR = 0.44, *p* = 0.04) [16]. Cohen et al. reported that 2.6% of black participants (*n* = 3,363) had nonsense mutations in PCSK9, which was associated with an 88% lower risk of CAD (*P* = 0.008) [42]. Analysis of the *CETP* gene revealed that protein truncation variant carrier status was associated with a reduced risk of CAD (OR = 0.70, *P* = 5.1 × 10 − 3) [25]. To our knowledge, no previous study has reported the relationship between rare variants in lipid metabolism genes and CAD in a Chinese Han cohort. However, there have been association studies of rare variants in other genes with CAD. Jia et al. sequenced nine exons of the *CPE* gene in 51 CAD patients, and no significant associations were found between rare variants and CAD [43]. Sequencing of *MEF2A* exon 11 revealed a rare 21-bp deletion in five CAD patients, indicating that this deletion might be a specific cause of CAD [44]. Wang et al. genotyped the rare variant rs34166160 in *NINJ2* and demonstrated that rs34166160 significantly confers risk of CAD [45].

**Fig. 1:**
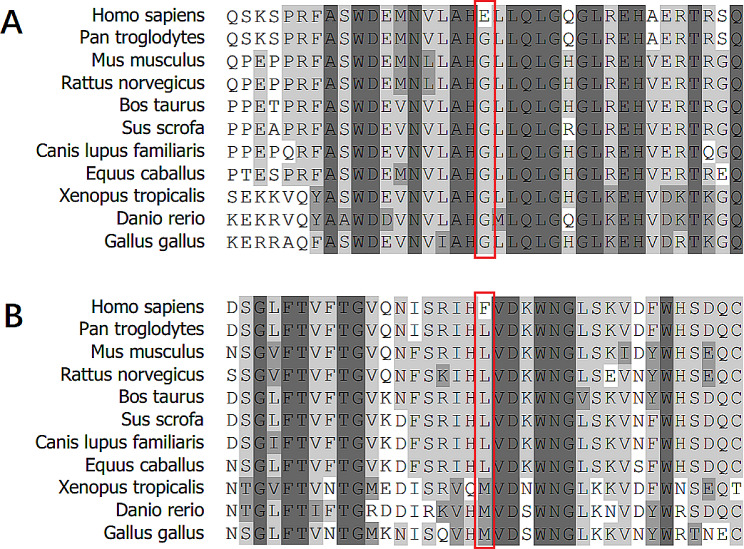
Protein sequence alignment across species. **A:** Amino acid sequence alignment of *ANGPTL4* across 11 species. The variant Gly47Glu is indicated by a red box. **B:** Amino acid sequence alignment of *SCARB1* across 11 species. The Leu233Phe variant is indicated by a red box

In the present study, a total of 33 nonsynonymous rare variants were identified. However, a gene-based SKAT-O test did not reveal an association between rare variants and CAD. We found two novel variants in the CAD cohort. One of them was a variant that introduces a missense variant in *ANGPTL4* (p.Gly47Glu), which was predicted to be deleterious by SIFT and probably damaging by PolyPhen-2. The other variant was a variant that introduces a missense variant in *SCARB1* (p.Leu233Phe), which was predicted to be tolerated by SIFT and possibly damaged by PolyPhen-2. Protein sequence alignment of the novel variants revealed that both variants affect residues highly conserved across multiple species (Fig. 1). *ANGPTL4* inhibits LPL activity and retards lipoprotein catabolism Previous studies have shown that carriers of the *ANGPTL4* variant are more likely to have lower triglyceride levels and higher HDL cholesterol levels than noncarriers are and are less likely to have CAD [13]. In this study, the HDL cholesterol levels (1.35 vs. 0.96 ± 0.23) of ANGPTL4 Gly47Glu variant carriers were greater than those of noncarriers, while the TG levels (1.33 vs. 1.59 ± 0.79) were lower. These results are consistent with those of previous studies [13]. Multiple studies have shown that missense variants in the *SCARB1* gene are associated with elevated HDL cholesterol levels. Zanoni et al. identified 3 heterozygous carriers and 1 homozygous carrier of p.Pro376Leu through targeted sequencing of *SCARB1* in 328 individuals with extremely high plasma HDL cholesterol levels, while the variant did not exist in 398 individuals with extremely low plasma HDL cholesterol levels. Association analysis revealed that carriers of the p.Pro376Leu variant have an increased risk of CAD. (OR = 1.79, *P* = 0.018) [30]. In this study, individuals carrying the *SCARB1* p.Leu233Phe variant had lower-than-average HDL cholesterol levels (0.85 vs. 0.96 ± 0.23).

In the ClinVar database, rs730882109 in LDLR was classified as “conflicting interpretations of pathogenicity”, and rs200727689 in LDLR was classified as “pathogenic/likely pathogenic”. Both of these variants were linked to familial hypercholesterolemia-1 (FHCL1) in the ClinVar database and identified only in the CAD cohort.

## Conclusion

In summary, we described a novel targeted NGS panel that included 12 lipid metabolism genes. One common missense variant, LIPC rs6083, was significantly associated with a reduced risk of CAD. Of all the rare nonsynonymous variants identified in this study, 18 existed only in the CAD group, 12 were identified only in the control group, and 3 were identified in both groups. However, none of the gene-based SKAT-O tests revealed an association between rare variants and CAD. We identified 33 nonsynonymous rare variants, including two novel variants, one located in the ANGPTL4 gene (p.Gly47Glu) and the other in the SCARB1 gene (p.Leu233Phe). This study suggests that targeted sequencing approaches can be used to discover common and rare variants that contribute to the aetiology of CAD risk and may lead to the discovery of novel pharmaceutical targets for disease prevention and treatment. However, this study has several limitations. (1) This assay was designed to detect single nucleotide variants and small indels, but larger indels or structural rearrangements were missed. (2) Whether these variants alter CAD risk remains unclear due to the lack of statistical power. A larger sample size is needed to increase the statistical power. (3) Determining the pathogenicity of novel variants by computational methods alone is difficult. Functional testing may help to clarify the impact of these variants. (4) As an ageing-related disease, CAD might develop in subjects in the control group in the future, leading to misclassification bias [46]. However, further studies are needed to validate these findings and explore these variations as potential pathogenic variants for CAD.

### Electronic supplementary material

Below is the link to the electronic supplementary material.

Supplementary Material 1

Supplementary Material 2

Supplementary Material 3

## Data Availability

All the data generated or analysed during this study are included in this published article [and its supplementary information files]. All the participants and/or their legal guardian(s) consented to the research process, producing the raw data, tables, and figures for publication. Patient names and patient images are not presented in this study. The datasets generated and/or analysed during the current study are available in the NCBI repository [https://www.ncbi.nlm.nih.gov/sra/PRJNA976403].

## Abbreviations

CAD: Coronary artery disease
NGS: Next-generation sequencing
