# Supplementary material for: Rare and common coding variants in lipid metabolism-related genes and their association with coronary artery disease
Source: BMC Cardiovasc Disord. 2024 Feb 9;24:97. doi: 10.1186/s12872-024-03759-5 (PMC10858582; doi:10.1186/s12872-024-03759-5)
Supplement: Supplementary file 3 — Supplementary Material 3 [file 12872_2024_3759_MOESM3_ESM.docx]

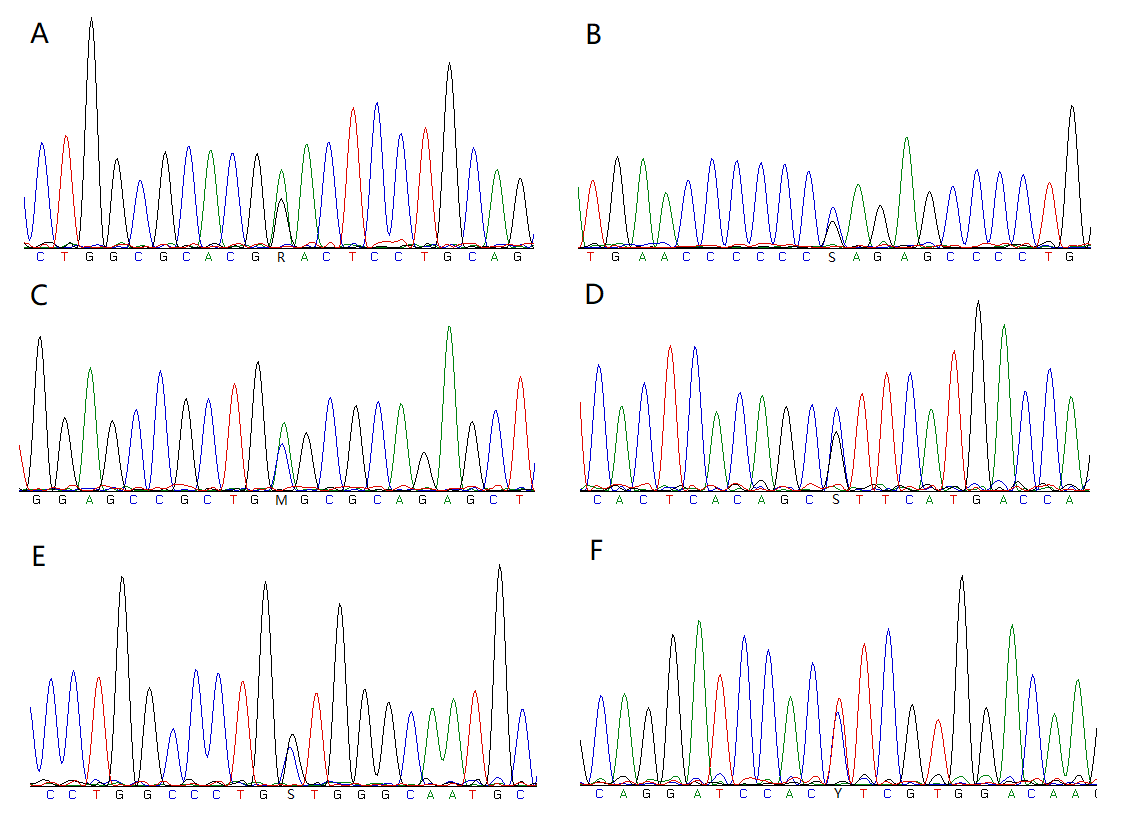


Supplementary Figure 1: Sanger capillary electrophoresis sequencing results of two novel variants and four novel alleles. A: novel variant, ANGPTL4 Gly47Glu; B: novel allele, APOA1 rs1254205437 G/C; C: novel allele, APOA1 rs1591330063 G/T; D: novel allele, APOA5 rs556600766 G/C; E: novel allele, CETP rs1460617147 C/G; F: novel variant, SCARB1 Leu233Phe.
